# DISSEMINATION AND IMPLEMENTATION OF AGE-INCLUSIVE MANAGEMENT STRATEGIES WITHIN INSTITUTIONS OF HIGHER EDUCATION

**DOI:** 10.1093/geroni/igae098.1435

**Published:** 2024-12-31

**Authors:** Brian Kaskie

**Affiliations:** University of Iowa, Iowa City, Iowa, United States

## Abstract

Institutions of higher education employ a greater proportion of persons over 65 relative to the general labor force. Age-Inclusive Management Strategies (AIMS) seeks to change the narrative about aging academics by shifting focus away from individuals and toward organizational efforts to adopt strategies designed to recruit, retain, and support the financial security and well-being of the growing population of aging faculty and staff. Using the University of Iowa as a case study, I recount efforts to analyze data concerning the productivity and retirement of aging employees, engage and activate institutional leadership to support implementation of policies and programs for aging faculty and staff, and the adoption of three management strategies. These strategies include: offering information and education about retirement and age-related issues such as estate planning and end of life care, increasing access to caregiver support programs, and modifying policies to facilitate access to varied retirement pathway option for faculty and staff alike. Such efforts have been used to validate why Iowa City and the state of Iowa are consistently recognized among the best places in America to grow older.

